# Play to Win: Action Video Game Experience and Attention Driven Perceptual Exploration in Categorization Learning

**DOI:** 10.3389/fpsyg.2020.00933

**Published:** 2020-05-13

**Authors:** Sabrina Schenk, Christian Bellebaum, Robert K. Lech, Rebekka Heinen, Boris Suchan

**Affiliations:** ^1^Institute of Cognitive Neuroscience, Clinical Neuropsychology, Neuropsychological Therapy Centre, Ruhr University Bochum, Bochum, Germany; ^2^Department of Children’s Pain Therapy and Paediatric Palliative Care, Faculty of Health, School of Medicine, Witten/Herdecke University, Witten, Germany; ^3^German Paediatric Pain Centre, Children’s and Adolescents’ Hospital, Datteln, Germany; ^4^Biological Psychology, Heinrich-Heine-University, Düsseldorf, Germany; ^5^Institute of Cognitive Neuroscience, Neuropsychology, Ruhr University Bochum, Bochum, Germany

**Keywords:** categorization learning, visual attention, perceptual processing, action video games, exception-based strategy, abstraction-based strategy

## Abstract

Categorization learning is a fundamental and complex cognitive ability. The present EEG study examined how much action video gamers differ from non-gamers in the usage of visual exploration and attention driven perceptual analyses during a categorization learning task. Seventeen healthy right-handed non-gamers and 16 healthy right-handed action video gamers performed a visual categorization task with 14 ring stimuli, which were divided into two categories. All stimuli had the same structure but differed with respect to their color combinations and were forming two categories including a prototype, five typical stimuli and one exception. The exception shared most similarities with the prototype of the opposite group. Prototypes and typical stimuli were correctly categorized at an early stage of the experiment, whereas the successful categorization of exceptions occurred later. The behavioral data yield evidence that action video gamers perform correct categorizations of exceptions earlier than non-gamers. Additionally, groups differed with respect to differential expressions of the attention related P150 ERP component (early perceptual analysis) and the N170 ERP component, which reflected differential processing demands for the stimulus material. In comparison to non-gamers, the analyses of the eye movements yield for action video gamers different, more central fixations possibly indicating covert peripheral processing. For both groups fixations as well as saccades decrease and in the case of exceptions, one of the two segments that are decisive for correct categorization shows higher fixation rates at the end of the experiment. These findings indicate for both groups a learning process regarding the stimulus material. Regarding the group differences, we interpret the results to indicate that action video gamers show a different stimulus exploration, use an enhanced early perceptual analysis of the stimulus material and therefore may detect changes in objects faster and learned the belonging of the stimuli to their categories in an earlier trial phase.

## Introduction

Categorization learning is a basic cognitive ability that determines everyday life. It is a cognitive function that helps us to assign objects as well as abstract concepts to specific categories (Smith and Minda, 1998; Zentall et al., 2008; Sloutsky, 2010). By using categorizations, hazards can be detected and successfully avoided (Smith et al., 2012), for example potentially toxic plants (Ashby and Maddox, 2005). Furthermore, the ability to categorize improves over the lifespan and is modulated by experiences (Quinn et al., 1993).

Based on the importance of categorization learning, it is not surprising that many theoretical models have been published (Ashby and Maddox, 2005; Ashby and Maddox, 2011; Lech et al., 2016; Schenk et al., 2016). For example, Cook and Smith (2006) concluded from their study that within the learning of categories a psychological shift from an early abstraction-based strategy to a later exception-based strategy takes place. This combination of strategies enables the categorization of realistic categories with a nuanced feature structure that consist of prototype-like stimuli and exceptions. In the current study we focused on the theoretical model of Cook and Smith (2006).

In general, cognitive flexibility as well as generalization processes are essential mechanisms that underlie categorization learning (Sloutsky, 2010). Focusing on category-typical features and ignoring all irrelevant information are necessary aspects of these processes. They are influenced by visual perception and supported by selective attention (Nosofsky, 1986; Thompson and Oden, 2000; Sloutsky, 2010). Visual attention is one of the main areas of investigation in categorization learning. But although selective attention is a part of many models of categorization learning (Nosofsky, 1986; Kruschke, 2001; Love et al., 2004), the effect of attention on categorization learning had not been investigated using the individual differences approach.

In our present work we compared the categorization performance of two groups who are thought to differ with respect to their attentional capacities. Based on previous studies, which have shown the positive impact of action video games on visual information processing (Green and Bavelier, 2006a, b, 2007; Feng et al., 2007), reaction times (Boot et al., 2008), multi-tasking (Green and Bavelier, 2006b; Strobach et al., 2012; Chiappe et al., 2013) and attention capacity (Green and Bavelier, 2003; Rosser et al., 2007; Hubert-Wallander et al., 2011; Bavelier et al., 2012), we compared action video gamers with non-gamers in a categorization learning task. Kühn et al. (2014) could show that even playing video games like Super Mario induces structural brain plasticity. These studies show that different types of video games have different effects on cognitive abilities and neurocognitive correlates. Besides, playing action video games appears to reduce gender differences in visual search and attention tasks (Stoet, 2011). Since playing action video games reduces gender differences (Stoet, 2011) and enhances selective visual attention and attention allocation (Rosser et al., 2007; Bavelier et al., 2012), it appears to be reasonable to make this comparison.

However, there are not many studies that shed light on the implications of these attentional benefits of action video gamers in categorization learning together with the underlying neurocognitive correlates. Categorization includes the combination of cognitive domains like attention, cognitive flexibility and visuospatial processing and visual perception. Palaus et al. (2017) reviewed the effect of video gaming on different cognitive functions in video gamers showing different effect of video gaming on each of these functions. The investigation of a task that combines these different cognitive functions, like it is realized during categorization, in video gamers provides a useful and important next step. Therefore, the investigation of visual attention during categorization related electrophysiological correlates appears to be of special interest. One of these event-related potentials (ERP) is the P150 component. This component has its focus at posterior electrode positions, is associated with a perceptual analysis of stimulus material and depends on the number of shared features within a category. The P150 reflects an early perceptual analysis and attention direction to specific stimulus segments (Johnson and Olshausen, 2003; Bigman and Pratt, 2004; Long et al., 2010). Additionally, we could already show in a former study (Schenk et al., 2016), that the P150 amplitude was higher in elderly subjects during categorization if compared to younger participants. Results of this study further suggest that the P150 amplitude reflects critical processes in categorization at least when exploring categorization using the paradigm introduced by Cook and Smith (2006). An additional ERP is the N170. It reflects visual expertise with non-face objects and early categorization related perceptual processes in the occipito-temporal cortex (Rossion et al., 2004). Inversion-effects (Diamond and Carey, 1986; Tarr and Gauthier, 2000), as well as behavioral (Gauthier and Tarr, 1997) and neuroimaging studies (Gauthier et al., 1999; Gauthier, 2000) indicated that N170 arises as a function of perceptual processing of expertise objects, such as Greebles (Tanaka and Curran, 2001; Rossion et al., 2002; Gauthier et al., 2003).

This study aimed to investigate how individual differences in visual exploration and attentional processing influence the performance and the electrophysiological correlates of categorization learning. For this, we used electroencephalography (EEG), eye-tracking, the same behavioral paradigm as Cook and Smith (2006) and compared two groups with different visual attention capacities. In reference to previous findings we compared action video gamers, who have an enhanced visual attention capacity, and non-gamers, who have a normal visual attention capacity. We expected that the behavioral results of Cook and Smith (2006) can be replicated. Furthermore, we expected that action video gamers use an enhanced perceptual analysis, detect relevant information faster and therefore show a faster and better learning performance. The faster detection of relevant information and the enhanced perceptual analysis should be accompanied by higher P150 amplitudes of action video gamers. Additionally, it is assumed that action video gamers exhibit less scattered and more center focused eye movements than non-gamers.

## Materials and Methods

### Participants

Seventeen subjects without action video game expertise (mean age 22.53 years; 3 male/14 female; non-gamers) and 16 subjects with high expertise in action video games (mean age 23.94 years; 14 male/2 female; action video gamers) participated in the current study. Participants with high action video game experience formed the group of the action video gamers. They were assigned to this group by fulfilling the criterion of playing a first-person shooter game for more than 20 h per week using a self-designed questionnaire (Supplementary Datasheet 1). The group of non-gamers consisted of subjects without any game experience playing not video games at all. All subjects were right-handed, neurologically healthy and had normal or corrected-to-normal vision. The neurological health and handedness were recorded via a self-report questionnaire. After a detailed explanation of the procedure all subjects gave informed written consent. The Ethics Committee of the Faculty of Psychology, Ruhr University Bochum, Germany approved the study.

### Stimuli and Task

**FIGURE 1:**
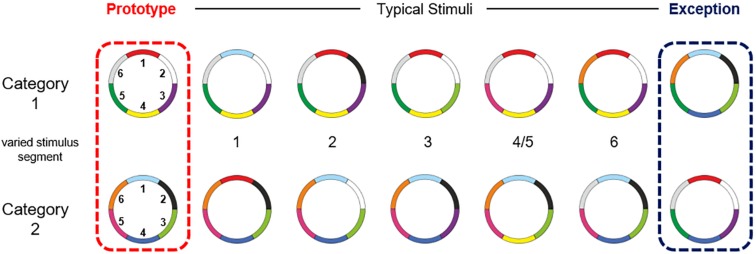
Overview of the stimulus material. The small numbers in the middle represent the stimuli segment (clockwise) on which the stimuli varied from the prototype. The stimuli of both categories shared an average of 3.88 colors within and 2.12 colors between the categories. They shared an average of 4.57 colors with their own prototype and 1.43 with the prototype of the other category (see also Schenk et al., 2016).

The participants performed an adapted version of the visual categorization task from Cook and Smith (Cook and Smith, 2006), with two categories and ring stimuli with six color features (768 × 768 pixels). These ring circles had a radius of 12.95° visual angle. Color features located at the outer side of the circle had a width of 1.74° visual angle. The stimuli of both categories had the same structure but differences in the combination of color features. Participants had no prior knowledge about the stimuli or categories. Both categories consisted of seven stimuli: one prototype, five typical stimuli and an exception. The typical stimuli shared five color features with the prototype of their own category. The exception shared five color features with the prototype of the other category (Cook and Smith, 2006; Schenk et al., 2016). An overview of the stimulus material, including the delineation of the two categories and the labeling of the stimulus types, as well as the numbering of the color features is shown in Figure 1. Subjects were not informed about the existence of exceptions. Since the exclusive usage of an implicit abstraction-based strategy would lead to errors in the categorization of the exceptions, the participants had to realize the existent of exceptions and had to explicit remember both exceptions, to correctly categorize all stimuli (Lech et al., 2016).

**FIGURE 2:**
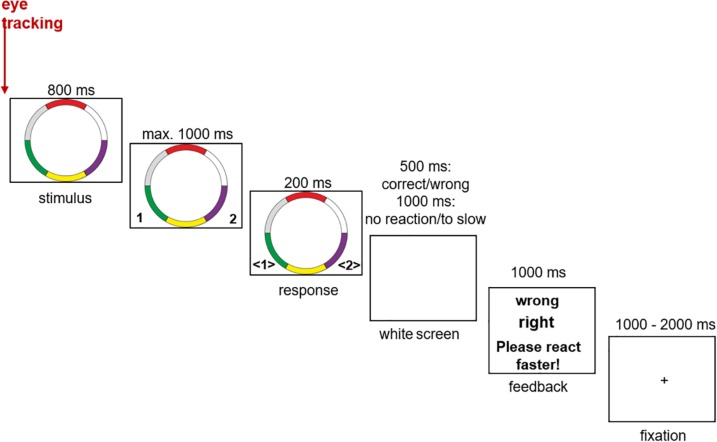
Experimental setup of the categorization task (see also Schenk et al., 2016).

In the center of a white background, the ring stimuli were presented with a screen resolution of 1024 × 768 pixels. The left and right CTRL keys were used as response buttons. Each button corresponded to one of the two categories. After the response the participants received immediate feedback (“right” or “wrong”). If the subjects react too slowly (more than 1.8 s after stimulus presentation), they were given an information via the feedback to react faster (“Please react faster!”). After the feedback presentation (1 s) a fixation cross was presented for a variable inter trial interval of 1 to 2 s before the next trial started. The learning process is taught through feedback. The experiment consisted of a total of 490 trials, divided into five blocks each, containing 98 that were presented in a random order (14 prototypes, 70 typical stimuli, and 14 exceptions). After every block the participants were allowed to take a short break (Figure 2).

### EEG Recording

The experiment was performed at the Ruhr University Bochum in an EEG laboratory using Presentation^®^ 16.2 software package^1^ (NeuroBehavioral Systems, Inc.). During the experiment, EEG was recorded from 30 electrodes according to the international standardized 10–20 system. For the recording a BrainProducts Amplifier (BrainAmp, BrainProducts, Munich, Germany) and the appropriate software package “BrainVision Recorder” were used with a sample rate of 500 Hz. A ground electrode was placed at FCz and the reference electrodes were placed at the mastoids. The impedances of the electrodes were kept below 5 kΩ.

### Eye-Tracking

Eye position data of participants’ right eye were captured during the stimulus presentation with a sampling rate of 500 Hz. For the recording of the fixations a video-based iView XTM Hi-Speed system (Senso Motoric Instruments, Berlin, Germany) was used. A calibration of the eye-tracker to the individual eye position of the participants was implemented at the beginning of each block (Figure 2).

### Data Analyses

The recorded EEG dataset was analyzed with the BrainVision Analyzer Software of Brain Products (BrainVision Analyzer, Package 2.0, Brain Products, Munich, Germany). A band-pass filter with cutoffs of 0.5 and 40 Hz (order 2; Time Constant = 0.3 s) were used. Blink artifacts and vertical eye movements were removed from the EEG data of each subject after individual independent component analyses (ICA). Components reflecting eye movements were removed from the EEG signal. Because of their similarity prototypes and typical stimuli were combined (prototypical stimuli). Segments for both stimulus types (prototypical stimuli and exceptions), followed by a baseline correction relative to 200 ms preceding the stimulus presentation were created. Trials with data points exceeding an absolute amplitude value of 150 μV were excluded by automatic artifact detections (see Supplementary Table 1 for the numbers of included EEG trials). Single subject averages were created for both stimulus types (prototypical stimuli, exceptions).

The P150 ERP component is related to early selective visual attention and has its focus at posterior electrode positions. In the categorization paradigm introduced by Cook and Smith (2006) it is a critical component for the categorization performance (Schenk et al., 2016). Similar to the study by Schenk et al. (2016), the analysis of the P150 component included data from the parietal-occipital electrodes (PO7, PO3, POz, PO4, PO8). The maximum positive peak amplitude within the corresponding time frame of 120 to 180 ms after stimulus presentation was defined as the P150 ERP amplitude. The amplitudes of action video gamers and non-gamers were compared for both stimulus types (prototypical stimuli and exceptions). The maximum negative peak amplitude within the corresponding time frame of 150 to 190 ms after stimulus presentation was defined as the Peaks of the N170 amplitude. The analysis of the expertise related N170 was based on data from the P7, PO7, P8, and PO8 electrodes. Peak amplitudes were determined automatically in a first step by the Vision Analyzer software. Visual inspection of this procedure was performed afterward to ensure correct peak detection.

The eye-tracking data were analyzed using MATLAB R2009a (MATLAB and Statistics Toolbox Release 2009a, The MathWorks, Inc., Natick, MA, United States). Fixations were visualized on the basis of heat maps. According to the structure of the stimuli, the ring stimuli were divided into eight areas: the seven color segments of the stimuli and the center of the stimuli. The center of each stimulus was defined as a circle around this center of with a radius of 8° visual angle (191 pixel). Percentages of the fixation rates and numbers of fixation were calculated for the area of all color segments in total and for the center of the stimuli. The differences in the fixation rates within and between the two groups were compared for prototypes and exceptions. Overall, differences between a central holistic processing and a stimulus segment driven processing were investigated. Additional analyses focused on specific color segments of the stimuli that were import for the correct categorization of exceptions. Apart from fixations, eye movements were also analyzed. More specifically, saccades were detected during the 800 ms stimulus presentation period, based on individual subjects’ eye position data in each trial. Changes in eye position were considered as saccades when the velocity of the change in eye position exceeded 40° visual angle per second and when the amplitude of the eye displacement was at least 1.5° visual angle. Saccade onset was determined as the point in time when the velocity exceeded the threshold of 40° visual angle per second.

For the statistical analysis of the behavioral data, prototypes and typical stimuli were combined to prototypical stimuli for the analysis. The focus of the analyses was on the percentage of correct responses as it reflects learning progress. Additionally, reaction times were analyzed within and between the groups. Two ANOVAs with repeated measures (including Greenhouse–Geisser procedure and Bonferroni correction with an alpha = 0.05) and the factor “stimulus” (prototypical stimuli; exceptions), the factor “block” (1 to 5), and the “group” factor (non-gamers; action video gamers) were applied. To resolve significant interactions two-tailed *t*-tests were applied. Sample size planning with G^∗^Power (G^∗^Power: Statistical Power Analyses; Faul et al., 2007), an α = 0.05, a statistical power of 95% and an assumed moderate effect of *f* = 0.25 yielded optimal sample sizes of *N* = 32 (*n* = 16 per group).

For the statistical analysis of the EEG data an ANOVA with repeated measures was calculated for the P150 amplitude over all five electrodes. Additionally, Greenhouse–Geisser and additional Bonferroni corrections (alpha = 0.05) were used. The ANOVA contained the factors “stimulus” (prototypical stimuli; exceptions), “block” (1 to 5) and “group” (non-gamers; action video gamers). To resolve significant interactions two-tailed *t*-tests were applied. The *a priori* determination of the sample size (G ^∗^ Power: Statistical Power Analyses, Faul et al., 2007) with the factor stimulus, the repeated measurement factor bock, the group factor, an α = 0.05, a statistical power of 95% and an assumed moderate effect of *f* = 0.25, gave an minimal sample size of *N* = 32 (*n* = 16 per group).

The first analysis of the fixation eye-tracking data investigated the fixation behavior of the stimuli (central with peripheral exploration and focused on stimulus segments). Sample size planning with G^∗^Power with an α = 0.05, a statistical power of 95% and an assumed moderate effect of *f* = 0.35 yielded for the eye-tracking data an optimal sample size of *N* = 30 (*n* = 15 per group). A Bonferroni-corrected repeated measures ANOVA with the factors “stimulus” (prototypes; exceptions), “fixation behavior” (central; stimulus segments), “block” (1; 5), and “group” (non-gamers; action video gamers) was computed to investigate the fixation rates. In case of sphericity violation, a Greenhouse–Geisser correction was used. The second, as well as the third analysis of the fixation data focused on the fourth and fifth stimulus segments. These analyses examined whether participants focused on specific stimulus segments that are important to correctly categorize the exceptions and their category membership. Both analyses included Bonferroni corrections with an alpha value of 0.05 and Greenhouse–Geisser procedures. The second repeated measure ANOVA was computed with the factors “stimulus” (prototypes; exceptions), “block” (1; 5), and the between-subject factor “group” (non-gamers, action video gamers). The analysis examined whether the subjects of both groups had higher fixation rates in the fourth stimulus segment in order to correctly classify category 2 exceptions. Apart from the fourth segment, exceptions of category 2 look similar to prototypical stimuli of category 1. A fixation of the fourth stimulus segment is required for a correct assignment of stimuli as category 2 exceptions. In a third step, another repeated measure ANOVA with the factors “stimulus” (prototypes; exceptions), “block” (1; 5), and “group” (non-gamers; action video gamers) investigated whether subjects looked more at the fifth stimulus segment in order to categorize exceptions of category 1 correctly. Apart from the fifth stimulus segment, exceptions of category 1 look similar to prototypical stimuli of category 2. A fixation of the fifth stimulus segment is necessary to assign stimuli correctly as exceptions of category 1. To further elucidate the eye-tracking data, two additional ANOVAs were performed. One ANOVA included the amount of fixation on each segment before decision. This ANOVA included the factors “stimulus (prototypes; exceptions), “block” (1; 5), and “group” (non-gamers; action video gamers) was performed. Another ANOVA included the same factors using the numbers of eye movements as dependent variable.

Furthermore, saccades were analyzed. The first analysis referred to the mean number of saccades that were performed during the first 800 ms of stimulus presentation. For each participant the mean number of saccades per trial was calculated for prototypes and exceptions in each of the five blocks. Then a repeated measure ANOVA with the factors “stimulus” (prototypes; exceptions), “block” (1; 5), and “group” (non-gamers; action video gamers) was performed. In a second step, an ANOVA with the same factors was applied to analyze the mean latency of the first saccade.

## Results

### Behavioral Data

**FIGURE 3:**
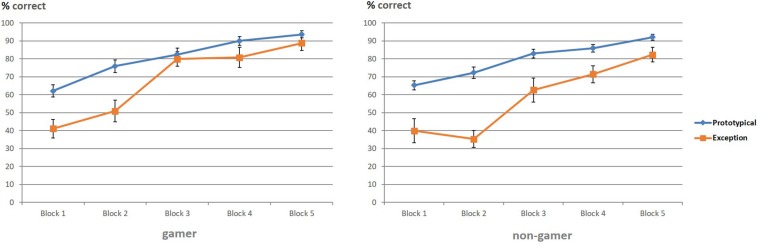
Correct categorizations of prototypical stimuli and exceptions (in percent).

The ANOVA for the percentage of correct responses yielded significant main effects for the factor “stimulus” (*F*_1,31_ = 78.24, *p* = 0.0001) and the factor “block” (*F*_4,86_ = 83.99, *p* = 0.0001). Additionally, the ANOVA showed a significant interaction of the factors “stimulus” and “block” (*F*_3,85_ = 5.83, *p* = 0.001). The following pairwise comparisons revealed for both groups more correct categorizations in the last blocks, especially for prototypical stimuli. The paired *t*-test showed that the interaction was based on the high number of correctly categorized prototypical stimuli compared to the low number of correctly categorized exceptions at the beginning of the experiment and the strong increase of correctly categorized exceptions in block 3 (Figure 3 and Table 1). To further analyze the accuracy changes over the five blocks, we calculated the difference between percentage correct responses for exceptions and prototypical stimuli. Differences between the blocks (1 vs. 2, 2 vs. 3, 3 vs. 4, 4 vs. 5) were analyzed using paired *t*-tests. Results yield evidence for significant differences only between block 2 and block 3 [*t*(32) = 4.17; *p* < 0.001]. All other results showed no significant differences (all *p* > 0.05).

**TABLE 1:** Percentage of correct categorizations separated for stimulus types and group.

	Gamer	Non-gamer
**Prototypical**		
Block 1	62.13 (13.24)	65.27 (9.68)
Block 2	75.89 (13.81)	72.27 (12.53)
Block 3	82.44 (13.92)	82.91 (8.76)
Block 4	89.96 (9.37)	85.92 (8.34)
Block 5	93.68 (7.02)	92.09 (5.83)
**Exception**		
Block 1	41.07 (20.45)	39.92 (27.44)
Block 2	50.89 (23.89)	35.29 (19.80)
Block 3	79.91 (16.54)	62.61 (27.49)
Block 4	80.80 (22.55)	71.43 (19.40)
Block 5	88.84 (16.28)	82.35 (16.58)

*Data are presented as mean ± SDM.*

Regarding group differences, the ANOVA showed a significant interaction between “stimulus” and “group” (*F*_1,31_ = 5.35, *p* = 0.02), which was based on the superior categorization performance of action video gamers for exceptions (Figure 3). The group difference regarding the superior categorization performance of action video gamers for exceptions corresponded to a strong effect size (*d* = 0.83). In view of the descriptive data, exploratory analyses were calculated to examine this “stimulus” and “group” interaction separately for each of the five blocks. According to these analyses, it seemed that the interaction effect between the factors “stimulus” and “group” was particularly enhanced in block two (*F*_1,31_ = 4.19; *p* = 0.049, *d* = 0.73) and three (*F*_1,31_ = 4.72; *p* = 0.03, *d* = 0.82; see Table 1 and Figure 3) with a superior categorization performance of action video gamers for exceptions.

**FIGURE 4:**
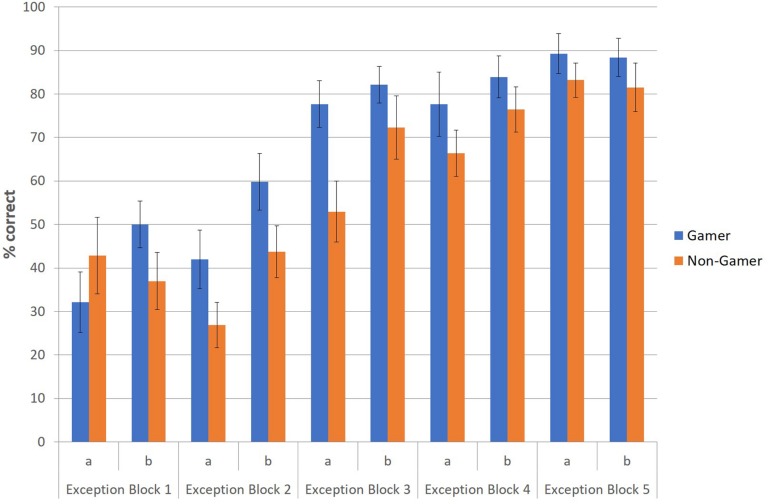
Correctly categorized exceptions (in percent) for the first (a) and second part (b) of each block.

To illustrate this advantage of action video gamers regarding the correct identification and categorization of exceptions, especially in the early stage of the paradigm, an additional figure was created to depict descriptively the correctly categorized exceptions (in percent) of both groups at the beginning (first part) and end phase (second part) for each of the five blocks (Figure 4). The figure shows that action video gamers categorized exceptions earlier and better than non-gamers throughout the entire experiment and especially at the beginning of each of the five blocks.

Additionally, an evaluative, descriptive comparison of behavioral data separated for male and female participants was performed. The descriptive comparisons of the behavioral categorization performance of male and female subjects showed that female gamers categorized better than female non-gamers and that male gamers performed better than male non-gamers. Besides, female gamers categorized better than male gamers and female non-gamers performed better than male non-gamers as well (see Supplementary Table 2 and Figures 1– 3). More detailed results can be found in the Supplementary Data.

**FIGURE 5:**
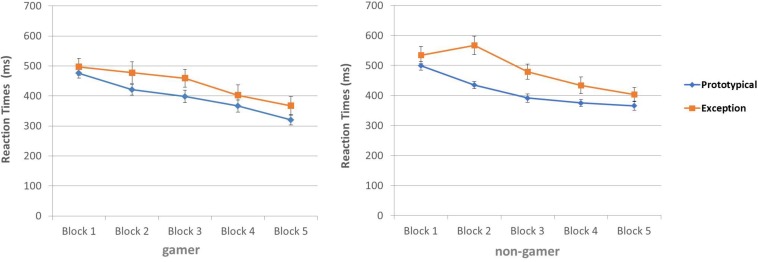
Reaction times (ms) for categorization of prototypical stimuli and exceptions.

The calculated ANOVA for the response times yielded evidence for a main effect of the factor “stimulus” (*F*_1,30_ = 36.05; *p* = 0.0001), a main effect of the factor “block” (*F*_4,120_ = 29.66; *p* = 0.0001), as well as an interaction of the factors “stimulus” and “block” (*F*_4,120_ = 2.72; *p* = 0.04). The three-way interaction did not reach significance. Reaction times separated for groups (gamers and non-gamers) are illustrated in Figure 5. The reaction times decreased during the experiment (block 1 to block 5) for both stimulus types. The largest difference seemed to be in block 3. However, participants categorized prototypical stimuli in total faster than exceptions (see Figure 5 and Supplementary Figures 4, 5 ).

### Eye-Tracking Data

**FIGURE 6:**
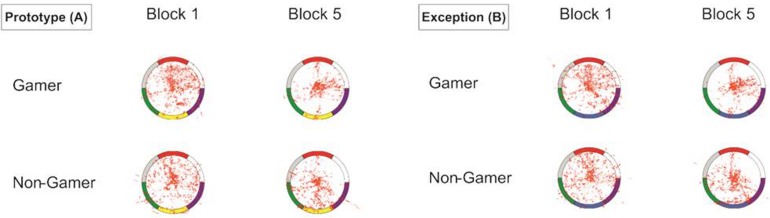
Heat map graphs of the eye-tracking data for both stimulus types (A = prototypes, B = exceptions).

The visualization of the eye-tracking data in heat maps showed for the start of the learning process scattered fixations over the whole stimulus (central and peripheral stimulus areas). However, the descriptive comparison also showed more focused eye movements for exceptions than for prototype stimuli. In contrast, at the end of the learning process, the eye movements were for both stimulus types more concentrated and focused on specific color segments. The descriptive group comparison showed that action video gamers exhibited less scattered and more center focused eye movements as compared to non-gamers, particularly apparent in the last block of the experiment (Figure 6).

The first statistical analysis on the fixation data investigated the fixation behavior in the first and the last block of the experiment (central fixation by exploring peripheral information and peripheral (focused) fixation on stimulus segments). The analysis showed a main effect for the factor “fixation” (*F*_1,31_ = 4.46, *p* = 0.04) and significant interactions of the factors “stimulus” and “fixation” (*F*_1,31_ = 7.08, *p* = 0.01) and of the factors “fixation” and “block” (*F*_1,31_ = 4.64, *p* = 0.03) as well as a significant interaction of the factors “stimulus,” “fixation,” and “group” (*F*_1,31_ = 4.34, *p* = 0.04). In general, the analysis showed higher fixation rates of the stimulus center (Table 2).

**TABLE 2:** Percentage of fixations for each group and both stimuli.

	Stimulus type	Central	Stimulus segments	*t*-score	*p*
**Gamer**	Prototypes	54.10 (5.43)	45.89 (5.43)	0.76	0.46
	Exceptions	71.68 (7.92)	28.33 (7.92)	2.74	0.02*
**Non-gamer**	Prototypes	52.04 (5.16)	47.96 (5.16)	0.40	0.70
	Exceptions	54.17 (5.08)	45.83 (5.07)	0.82	0.42

*t*-scores and *p*-values refer to the statistical comparisons of central and stimulus segment-based fixations within the stimulus types. Data are presented as mean ± SEM; *p ≤ 0.05.

**FIGURE 7:**
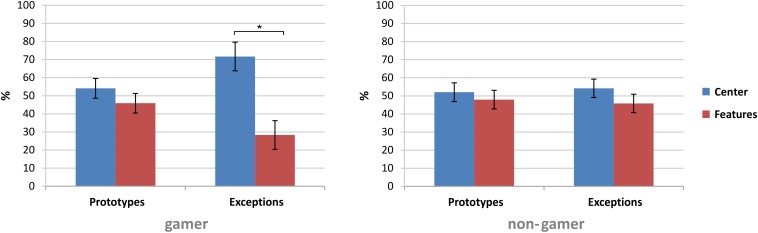
Group comparison of the fixation rates on the six stimulus segments and the stimulus center of prototypes and exceptions. All data are presented in percent as mean ± SEM; *p ≤ 0.05.

Subsequent paired comparisons revealed in the first block in total more fixations on the stimulus segments [*t*(32) = 2.13, *p* = 0.04, *d* = 0.37] and in the last block more fixations on the stimulus center [*t*(32) = −2.13, *p* = 0.04, *d* = 0.37]. Furthermore, the subsequent analyses yielded only for exceptions higher fixation rates of the stimulus center [exceptions: *t*(32) = 2.63, *p* = 0.01, *d* = 0.46; prototypes: *t*(32) = 0.83, *p* = 0.42]. In comparison to exceptions, the analysis showed for prototypes more fixations on the six stimulus segments [*t*(32) = 2.47, *p* = 0.01, *d* = 0.43]. Further subsequent analyses to resolve the interaction of the factors “stimulus,” “fixation,” and “group” revealed within the stimulus types that only action video gamers showed higher fixation rates on the center of exceptions than on the six stimulus segments of exceptions [*t*(15) = 2.74, *p* = 0.02, *d* = 0.69; see Table 2 and Figure 7].

The second statistical analysis of the fixation data, which focused on the fourth stimulus segment of the stimuli of the first category, did not yield significant results. However, the third analysis, which investigated the fixation rates on the fifth stimulus segment for stimuli from category 2, showed a significant interaction of the factors “stimulus” and “block” (*F*_1,31_ = 7.38, *p* = 0.01). Subsequent paired comparisons revealed for the last block with a small effect size significant more fixations on the fifth stimulus segment of exceptions [*t*(32) = −2.55; *p* = 0.01, *d* = 0.44].

A further analysis on the fixation data of the 4^th^/5^th^ segment was performed by combining the data from categories 1 and 2. The analysis focused on the idea that fixation on these segments should be observed also in the non-exception trials reflecting some performance control. Results of this analysis yielded neither significant main effects nor a significant interaction (all *p* > 0.05).

Concerning saccadic eye movements, the ANOVA on the mean number of saccades per trial yielded a main effect of block (*F*_4,124_ = 9.07; *p* = 0.002). The number of saccades decreased from the beginning to the end of the experiment (see Table 3) and some participants did not perform saccades at all during the first 800 ms of stimulus presentation toward the end of the experiment, which is in line with the pattern more fixations in the stimulus center at the end of the experiment that was described above. None of the other main effects and interactions for the number of saccades per trial reached significance (all *p* > 0.30). For the subsequent analysis on the mean latency of the first saccade those participants were excluded who did not perform saccades in at least three trials per block and condition. The sample for this analysis thus consisted of 10 gamers and 14 non-gamers. Again, a significant effect of block (*F*_4,88_ = 8.28; *p* < 0.001) was the only significant effect (for the remaining main effects and interactions all *p* > 0.28). The block main effect indicated an increase in saccade latencies during the course of the experiment (see Table 4), which is in line with the reduced number of saccades that were performed during stimulus processing. Both analyses on saccadic eye movements were repeated considering prototypes and typical stimuli combined in one condition and then compared with exceptions. The pattern of findings for these analyses was comparable with the pattern reported for prototypes vs. exceptions.

**TABLE 3:** Mean number of saccades per trial for prototypical and exception stimuli in blocks 1 to 5 in gamers and non-gamers (SD in brackets).

	Gamer	Non-gamer
**Prototypical**		
Block 1 Block 2 Block 3 Block 4 Block 5	2.93 (1.79) 1.98 (0.94) 1.41 (1.17) 1.36 (1.08) 1.17 (1.09)	3.18 (3.35) 1.68 (0.91) 1.75 (1.16) 1.66 (0.99) 2.11 (1.79)
**Exception**		
Block 1 Block 2 Block 3 Block 4 Block 5	2.74 (1.23) 1.96 (1.06) 1.39 (1.13) 1.39 (1.11) 1.18 (1.18)	3.04 (2.69) 1.87 (1.00) 1.95 (1.27) 1.50 (0.81) 1.79 (1.02)

*Data are presented as mean ± SDM.*

**TABLE 4:** Mean latencies of the first saccade per trial (in ms, SD in brackets) for prototypical and exception stimuli in blocks 1 to 5 in gamers and non-gamers.

	Gamer	Non-gamer
**Prototypical**		
Block 1 Block 2 Block 3 Block 4 Block 5	244.38 (59.40) 285.44 (43.64) 335.28 (99.08) 302.99 (92.75) 339.08 (69.48)	277.61 (56.22) 289.21 (79.27) 325.87 (97.99) 321.42 (84.58) 325.55 (101.58)
**Exception**		
Block 1 Block 2 Block 3 Block 4 Block 5	261.91 (48.43) 267.85 (41.46) 349.73 (94.41) 325.54 (82.34) 311.62 (65.29)	284.99 (63.62) 275.81 (91.55) 306.47 (71.37) 316.31 (84.40) 327.67 (76.00)

*Note that these data are based on a subsample of participants who performed saccades in at least three trials per condition and block. Data are presented as mean ± SDM.*

A further ANOVA including the fixation on the segments before decision showed a significant main effect for the factor “block” (*F*_4,124_ = 13.09, *p* < 0.001). Fixation rates decreased over the five blocks.

### EEG Data

**FIGURE 8:**
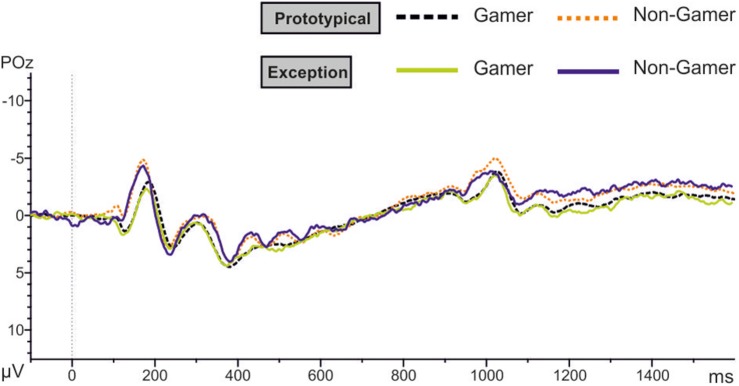
EEG results at POz position for both stimulus types.

The results of the P150 amplitude analysis showed a significant group effect with a strong effect size (*F*_1,31_ = 5.73, *p* = 0.02, *d* = 0.86). Action video gamers generally exhibited higher P150 amplitudes than non-gamers for both stimulus types (see Table 5 and Figure 8). The descriptive comparisons of the P150 amplitudes showed that male as well as female gamers had higher P150 amplitudes than male and female non-gamers (see Table 5).

**TABLE 5:** Analysis results of the P150 amplitudes (μV).

	Gamer	Non-gamer
**Prototypical**		
**All**	**2.58 (1.20)**	**0.81 (2.63)**
Men	2.71 (1.18)	0.86 (1.83)
Woman	1.63 (1.22)	0.80 (2.83)
**Exception**		
**All**	**3.29 (1.80)**	**1.39 (2.96)**
Men	3.42 (1.89)	1.91 (1.62)
Woman	2.43 (0.18)	1.28 (3.21)

*Data are presented as mean ± SDM.*

In addition, the analysis yielded for the N170 amplitude a significant between-subject effect (*F*_1.00,31.00_ = 7.70, *p* ≤ 0.01). The amplitude is more negative for non-gamers than for action video gamers (Table 6 and Figure 8). Over all participants the analysis yielded a main effect for the factor electrode (*F*_1.92,59.64_ = 4.63, *p* = 0.05). The PO7 and the PO8 amplitudes are more negative than the others (see Table 6 and Figure 8). Separated analyses of the both groups showed different profiles. Only for the non-gamers there was a significant effect for the factor electrode (*F*_1.91,30.48_ = 3.70, *p* = 0.05). For action video gamers the analysis yielded no significant main effects or interactions. Furthermore, the analysis of the N170 latency revealed an interaction between the factors electrode and group (*F*_1.73,53.64_ = 4.89, *p* ≤ 0.05), such as the factors stimulus and electrode (*F*_1.97,60.92_ = 3.28, *p* = 0.05). Action video gamers had descriptively higher latencies at the right side (*F*_1.00,31.00_ = 1.28, *p* = 0.27; see Table 6) and non-gamers showed almost significant higher latencies at the left side (*F*_1.00,31.00_ = 3.88, *p* = 0.058). For exceptions non-gamers showed a significant higher PO7 latency (*F*_1.00,31.00_ =4.49, *p* = 0.05), meanwhile action video gamers had higher latencies at the P8 position for prototypical stimuli (*F*_1.00,31.00_ = 3.72, *p* = 0.06). Besides, the P7 and PO7 latencies were descriptively shorter for prototypical stimuli and the P8 and PO8 latencies were descriptively shorter for exceptions (all *p* ≥ 0.10; see Table 6).

**TABLE 6:** Means and standard deviations of the N 170 amplitudes (μV) for electrode position P7 and P8.

	Gamer		Non-gamer	
	
	P7	P8	P7	P8
**Prototypical**	−0.61 (0.78)	−1.12 (1.85)	−2.06 (1.98)	−3.10 (3.2)
**Exception**	−0.98 (0.96)	−0.76 (1.84)	−2.25 (2.33)	−3.22 (3.68)

*Data are presented as mean ± SDM.*

## Discussion

The behavioral results are in line with the findings of Cook and Smith (2006), showing that both groups categorized prototypical stimuli in prior blocks and with shorter reaction times than exceptions. At the beginning of the experiment prototypical stimuli were better categorized than exceptions, but in the third block the number of correctly categorized exceptions increased (Lech et al., 2016; Schenk et al., 2016). In line with this, the subsequent analyses of accuracy changes over the five blocks yield evidence for significant differences only between the second and third block. Furthermore, participants categorized prototypical stimuli in total faster than exceptions, but highest differences in the reaction times for the two stimulus types could be detected in the third block. These results may be due to the different learning strategies required for the stimulus types or the need to explicitly remember exceptions. However, the use of special or different learning strategies cannot be clearly demonstrated.

The analyses and illustrations of the behavioral learning data showed a significant group-stimulus interaction, which was based on the superior categorization performance of action video gamers for exceptions, especially in the second and third block. Regarding the categorization performance of exceptions, action video gamers additionally showed a better categorization performance at the beginning of each block throughout the experiment. Nevertheless, the advantage of action video gamers is balanced toward the end of the experiment. It seemed that non-gamers need more trials to correctly categorize the exceptions.

Based on the stimulus-type-dependent learning curves, differences in the eye-tracking data with respect to the stimulus type and between the beginning and the end phase of the learning process might have been expected. Especially in terms of the two stimulus segments that are critical for categorizing exceptions, higher fixation rates during the categorization of exceptions were expected. The visualization of the eye-tracking data showed in the first block in total more fixations on the stimulus segments and in the last block more fixations on the stimulus center. However, the statistical analyses showed for all eye-tracking measures except one (center vs. periphery fixations) no differences between action video gamers and non-gamers. The analyses mainly yielded only for exceptions higher fixation rates of the stimulus center. For both groups fixation rates decreased over the five blocks. Furthermore, the number of saccades decreased from the beginning to the end of the experiment, which is in line with the pattern more fixations in the stimulus center at the end of the experiment. In addition, an increase in saccade latencies during the course of the experiment was observed. As expected in the case of exceptions, one of the two segments that are decisive for correct categorization shows higher fixation rates at the end of the experiment. These findings indicate for both groups a learning process regarding the stimulus material. It is possible that in an early learning phase, both groups tried to learn the stimuli based on their different color features and showed more fixations on the stimulus segments, while in the last block they showed more fixations on the stimulus center. The participants had learned the stimuli and did not need to explore them entirely anymore.

In general, only action video gamers showed higher fixation rates on the stimulus center for exceptions. In comparison to non-gamers, action video gamers showed different, more central fixations possibly indicating covert peripheral processing. This might also be explained by the special design of the stimuli used in this study. It can be hypothesized that this advantage of video game players might vanish if critical stimuli were presented in the center. On the other side it might be the case, that action video gamers show even in an experiment with such stimuli better performance as they might be faster in capturing visual information *per se*. The critical factor for such an advantage might be the processing of especially complexity of the stimuli. Stimuli with low information content or complexity might lead to a ceiling effect in performance independently whether the participants are action video gamers or not. Additionally, regarding fixation rates, it may be possible that the stimulus presentation times were too short to ensure a clear and detailed analysis of the stimuli. Furthermore, there was no clear instruction to fixate critical stimulus segments. As no group effect or interaction was observed for the eye tracking data, central fixation with possible peripheral exploration and processing of the information might reflect best the advantage of video game player in the current experiment with no changes over time. Nevertheless, the eye-tracking data yielded no clear information about the strategy used by the participants during categorization in this experiment. Unfortunately, this explanation is statistically not supported by the results.

Moreover, the analyses of EEG Data showed that groups differed with respect to differential expressions of the attention related P150 ERP component (early perceptual analysis). Action video gamers generally exhibited higher P150 amplitudes than non-gamers for both stimulus types. The higher P150 amplitudes of action video gamers may be an indicator for an early and more enhanced perceptual analysis of the stimulus material This early perceptual analysis maybe due to their video game experience, but, above all, is more useful for the categorization of exceptions, particular at the beginning of the learning paradigm. Therefore, it could facilitate a faster learning of exceptions with no changes over blocks.

We have shown in a recent study (Schenk et al., 2016) that it might be speculated whether the P150 amplitude is a critical EEG component which reflects the effort of an attention driven perceptual analysis used for categorization in general, especially in the task used in both studies. The study of Schenk et al. (2016) used the same categorization paradigm and compared the categorization performances as well as the P150 amplitudes of elderly and young subjects to investigate age-dependent differences. In contrast to young subjects, elderly subject showed a worse categorization performance in combination with a higher P150 amplitudes. Taken the results of both studies together, it seems that the P150 can be found with better and worse categorization performance. One possible interpretation of this is that action video gamers, as well as elderly subjects, used an enhanced early perceptual analysis, which requires a higher level of attention effort. It seems likely that elderly subjects need this enhanced attention driven early perceptual analysis to compensate their cognitive decline (e.g., in memory functions) in order to be able to learn the categories at all. Overall, it seems to be more difficult for elderly subjects to learn the explicitly remembered exceptions. It might be speculated whether the performance of elderly subjects might be even worse if the compensating P150 effect and its underlying process might have not been available. In contrast, the increased P150 amplitudes of action video players are more likely explained due to a training effect. Action video players have to perceive and analyze the environment within their games, including the smallest features, very quickly. This necessary early perceptual analysis requires high attention efforts in order to be able to react quickly. Action video gamers are trained in the detection of relevant stimulus information and may thereby use a more enhanced perceptual analysis, which is in this case especially important for the learning of exceptions. A further interpretation might be that for action video gamer the high P150 amplitude reflects their state during categorization based on their experience. In contrast, elderly subjects have to make an effort to achieve this state as a compensatory mechanism. Missing experience with action video gaming and cognitive decline might therefore not lead to the use of these processes that are reflected by the P150. In general, it has to be pointed out, that these are interpretations based on the results of these two studies.

Meanwhile, the significant group difference in the N170 amplitude might reflect group specific differences in perceptual process. It has been shown, that N170 amplitudes differs in the case of visual expertise (Rossion et al., 2004). Present results showed that non-gamers showed higher N170 amplitudes. This result suggests that the N170 is not exclusive to faces. It may further be speculated whether the higher N170 amplitude might reflect increased processing demands in the non-gamers as suggested by findings from Rossion et al. (1999) for faces. In contrast, lower N170 amplitudes in action video gamers could be due to a lower demanding for action video gamers in the current task.

Taken these findings together multi fold differences between action video gamers and non-gamers have been found which might support the idea of advantages for action video gamers in categorization learning, particular for exceptions and at an early learning stage. Similar to the study of West et al. (2015) the findings of the current study may be attributed to a more efficient direction in attention and an increased perceptual system of action video gamers that helped them to resolve the learning tasks. While we did find differences between the fixations and ERPs of action video game players and non-video game players, these differences did not always change as a function of block or exemplar type, making it difficult to link them to categorization specifically. The fixation group differences (central vs. peripheral) did interact with stimulus type, but the ERP differences were only manifested as main effects of group. Therefore, these results have to be considered independently and reflect each an individual factor that seems to be relevant for categorization. West et al. (2015) showed in their study that in contrast to non-gamers, it seemed that action video gamers showed an enhanced counting and remembering of specific sequences, features and target locations. This enhanced counting and remembering of specific features and locations could have given the action video players in the present study advantages in particular when learning the exceptions. It is possible that action video gamers can generate a more explicit knowledge of the exceptions through this different type of stimulus exploration and processing and can therefore remember them better.

However, it should be noted that the results of the current study only provide a first indication of potential group differences between the categorization performances of action video gamers and non-gamers. Different types of video games may lead in different impact (e.g., Green and Bavelier, 2003; Krishnan et al., 2013). A differentiated consideration of gaming varieties is necessary in order to be able to make concrete statements about the effects.

Another limiting factor of the current study is the heterogeneous sample of participants with an unequal gender distribution. Exploratory analyses suggest that women irrespective whether they were action video gamers or non-gamers outperform men in categorization (Supplementary Figures 1–3). This is interesting as other studies have shown that video games can lead to a reduction of gender differences in similar tasks (Subrahmanyam and Greenfield, 1994; Feng et al., 2007; Stoet, 2011). Based on the small sample size, this effect has to be seen very cautious. Separate statistical analyses with respect to gender are not meaningful. Further gender-balanced training studies with an intervention and control group employing a within-subjects design with pre- and post-tests are needed to pinpoint the exact effects of action video game experience on categorization learning and to exclude other personal factors.

## Data Availability Statement

The datasets generated for this study are available on request to the corresponding author.

## Ethics Statement

The studies involving human participants were reviewed and approved by Ethics Committee of the Faculty of Psychology, Ruhr University Bochum, Germany. The patients/participants provided their written informed consent to participate in this study.

## Author Contributions

SS conceptualized and designed the study, carried out the data analyses and interpreted data, drafted the initial manuscript and reviewed and revised the manuscript. CB analyzed the eye tracking data and wrote parts of the manuscript and reviewed it for important intellectual content. RL supervised data collection and critically reviewed the manuscript for important intellectual content. RH carried out additional data analyses of the eye-tracking data and reviewed the method section of the manuscript. BS conceptualized and designed the study, supervised data collection, interpreted data and reviewed the manuscript for important intellectual content. All authors approved the final manuscript as submitted and agreed to be accountable for all aspects of the work.

## Conflict of Interest

The authors declare that the research was conducted in the absence of any commercial or financial relationships that could be construed as a potential conflict of interest.

## Footnotes

1 http://www.neuro-bs.com
