# Supplementary material for: Play to Win: Action Video Game Experience and Attention Driven Perceptual Exploration in Categorization Learning
Source: Front Psychol. 2020 May 13;11:933. doi: 10.3389/fpsyg.2020.00933 (PMC7239510; doi:10.3389/fpsyg.2020.00933)
Supplement: Supplementary file 1 [file Data_Sheet_1.pdf]

SUPPLEMENTARY MATERIALS

Gender related Analysis

The calculated ANOVA for the response times yielded evidence for a main effect of the factor "stimulus" ($F_{1,30}$ = 36.05; p = 0.0001), a main effect of the factor "block" ($F_{4,120}$ = 29.66; p = 0.0001), as well as an interaction of the factors "stimulus" and "block" ($F_{4,120}$ = 2.72; p = 0.04). The three-way interaction did not reach significance. Reaction times separated for groups (gamer and non-gamer) and sex are illustrated in Figure 6. The reaction times decreased during the experiment (block 1 to block 5) for both stimulus types. The largest difference seemed to be in block 3. However, participants categorized prototypical stimuli in total faster than exceptions (see Figure 6).

FIGURE LEGEND - Supplements

Figure 1. Correct responses (in percent) for categorization of prototypical stimuli and exceptions separated for female and male gamer.

Figure 2. Correct responses (in percent) for categorization of prototypical stimuli and exceptions separated for female and male non-gamer.

Figure 3. Scatterplots of categorization performance separated for group and gender.

Figure 4. Reaction times (ms) for categorization of prototypical stimuli and exceptions of female and male gamer.

Figure 5. Reaction times (ms) for categorization of prototypical stimuli and exceptions of female and male non-gamer.

TABLES - Supplements

Table 1. EEG trials included in the analyses (mean, SD) and removed after artefact correction (mean, SD) separated for each condition and group.

	Prototypical Stimuli		Exception	
	Included	removed	Included	Removed
**Gamer**	405 (14.7)	2.6 (2.0)	67.8 (3.3))	0 (0)
**Non-Gamer**	379 (36.4)	5.2 (19.9)	63.7 (6.5)	0.11 (0.4)

Data are presented as mean± SDM.

Table 2. Gender-distributed correct categorization performance (%) of gamers and non-gamers.

	Gamer		Non-Gamer	
	**Men**	**Women**	**Men**	**Women**
**Prototypical**				
Block 1	62.84 (12.91)	57.14 (20.20)	92.86 (2.38)	65.56 (9.87)
Block 2	74.23 (13.86)	87.50 (7.58)	80.56 (5.37)	70.49 (13.02)
Block 3	80.19 (13.41)	98.21 (0.84)	87.30 (4.51)	81.97 (9.27)
Block 4	92.77 (7.06)	95.24 (6.73)	90.08 (2.75)	85.03 (8.92)
Block 5	89.20 (9.64)	100.00 (0.00)	63.89 (10.67)	91.92 (6.39)
**Exception**				
Block 1	41.33 (19.65)	39.29 (35.36)	69.05 (16.50)	33.67 (25.44)
Block 2	53.06 (24.70)	35.71 (10.10)	26.19 (8.25)	37.24 (21.19)
Block 3	79.59 (17.69)	82.14 (5.05)	33.33 (39.34)	68.88 (21.26)
Block 4	79.08 (23.52)	92.86 (10.10)	57.14 (25.75)	74.49 (17.43)
Block 5	87.24 (16.85)	100.00 (0.00)	71.43 (14.29)	84.69 (16.54)

Data are presented as mean± SDM.
