# Supplementary material for: Play to Win: Action Video Game Experience and Attention Driven Perceptual Exploration in Categorization Learning
Source: Front Psychol. 2020 May 13;11:933. doi: 10.3389/fpsyg.2020.00933 (PMC7239510; doi:10.3389/fpsyg.2020.00933)
Supplement: Supplementary file 2 [file Data_Sheet_2.pdf]

Screening Questionnaire

Please enter your test person code (VPN code)!

It is composed of a capital G, the first letter of your first and last name, your month and year of birth. **Example:**

Sarah Mustermann, born on 19.07.85 = GSM0785

Date of birth: ______________ **Gender:** ☐ male ☐ female

Average action video game consumption per week

☐ ≤ 4h ☐ ≤ 8h ☐ ≤15h ☐ > 20h

Handedness:

☐ right ☐ left

What educational qualifications do you have?

☐ Hauptschulabschluss
☐ Realschulabschluss
☐ Abitur
☐ Technical college/university degree
☐ Other

What is your current occupation?

☐ trainee
☐ (psychology) student
☐ PhD student
☐ working
☐ looking for work
☐ Pensioner

Are you aware of any previous neurological disorders? ☐ yes ☐ no

If so, which one: ____

Are you aware of any previous psychological illnesses? ☐ yes ☐ no

If so, which one: ____

Are you currently taking any medication (including hormonal contraceptives)? ☐ yes ☐ no

If so, which ____

Do you need a visual aid?

☐ no ☐ yes _____ **Diopters** If yes, balanced? ☐ yes ☐ no

Are you (currently) taking illegal drugs?

☐ never ☐ rarely ☐ often ☐ very often
