# Supplementary figures and images for: Play to Win: Action Video Game Experience and Attention Driven Perceptual Exploration in Categorization Learning
Source: Front Psychol. 2020 May 13;11:933. doi: 10.3389/fpsyg.2020.00933 (PMC7239510; doi:10.3389/fpsyg.2020.00933)

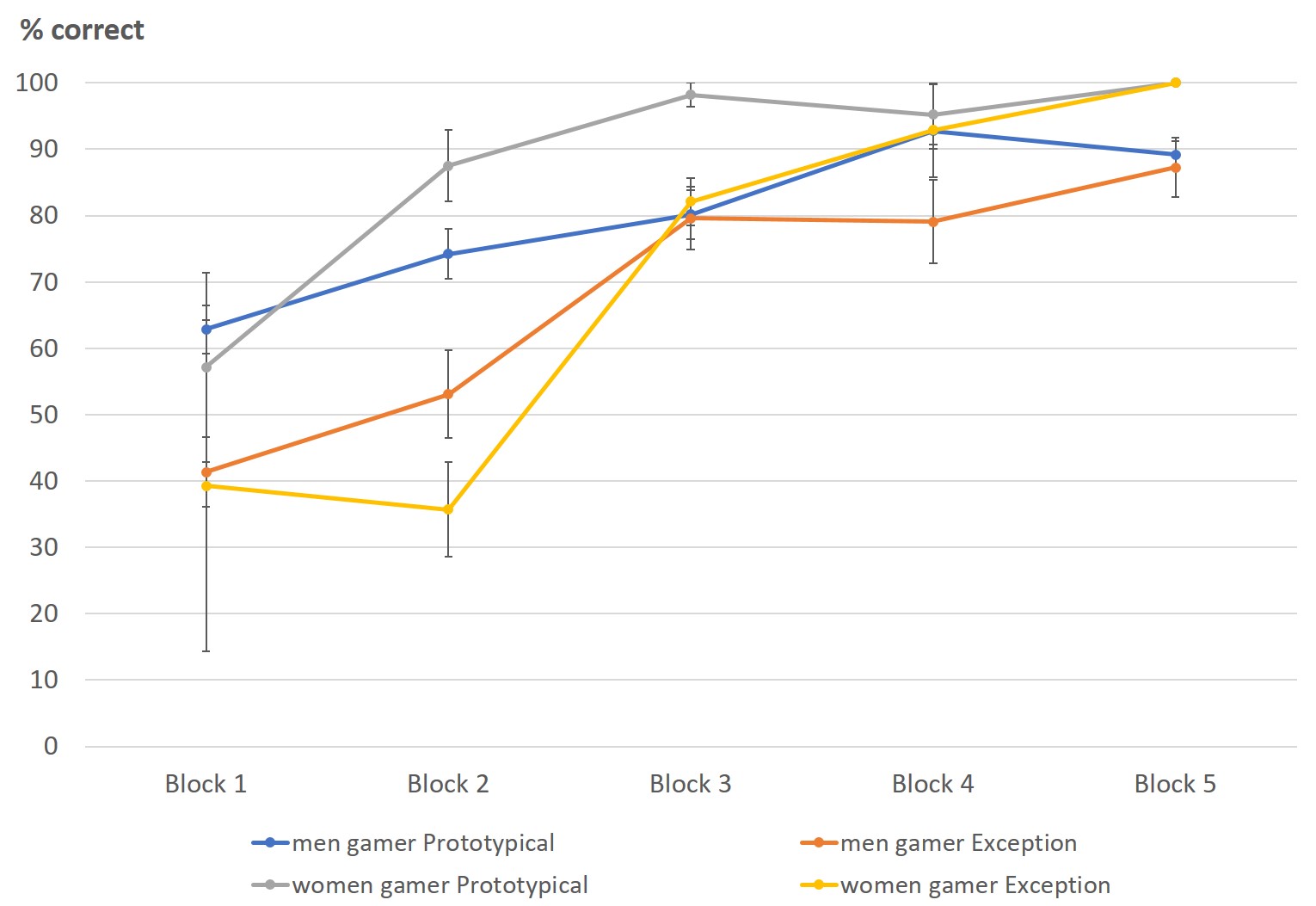

Supplement: Supplementary file 3 [file Image_1.JPEG]

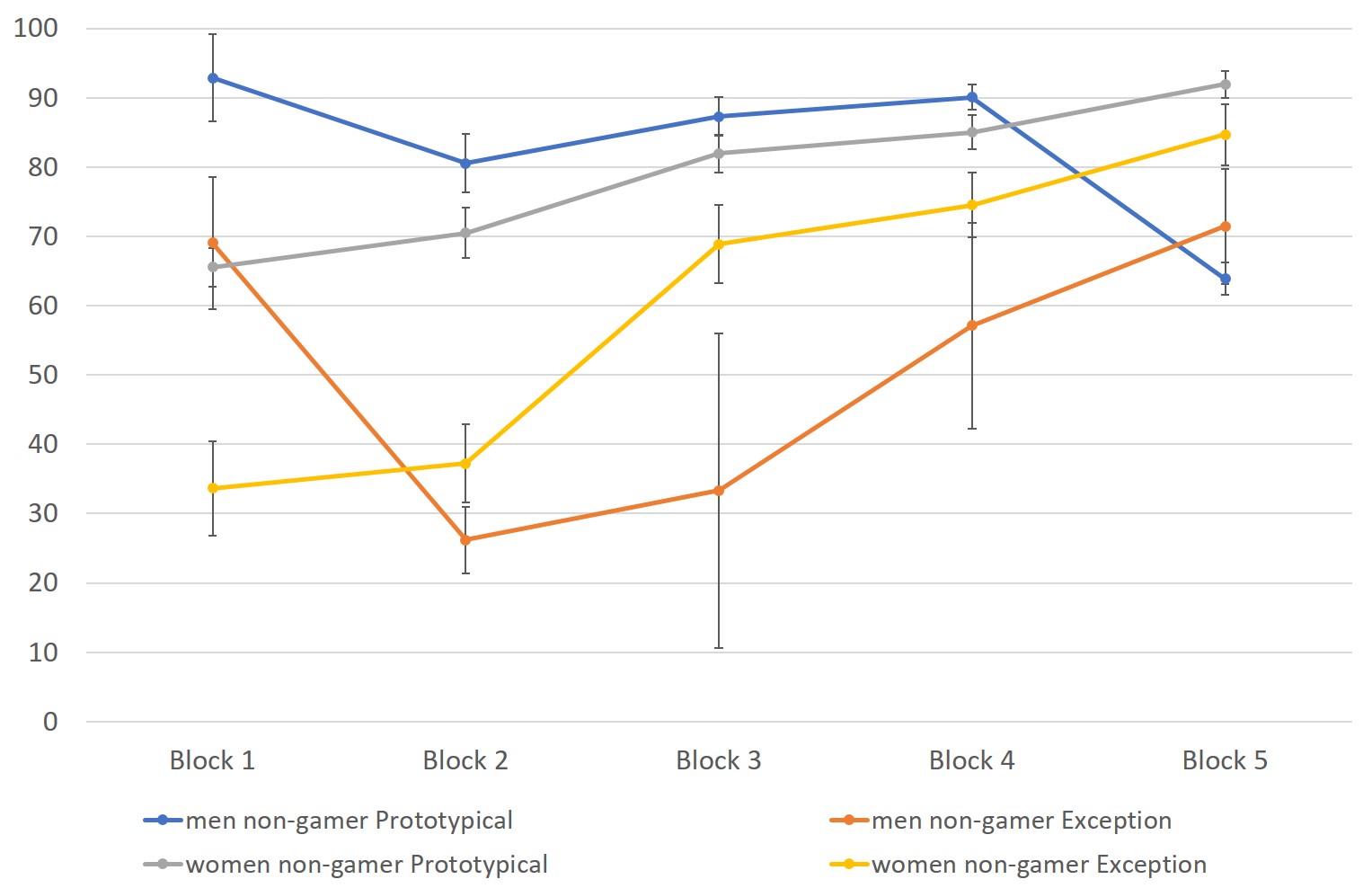

Supplement: Supplementary file 4 [file Image_2.JPEG]

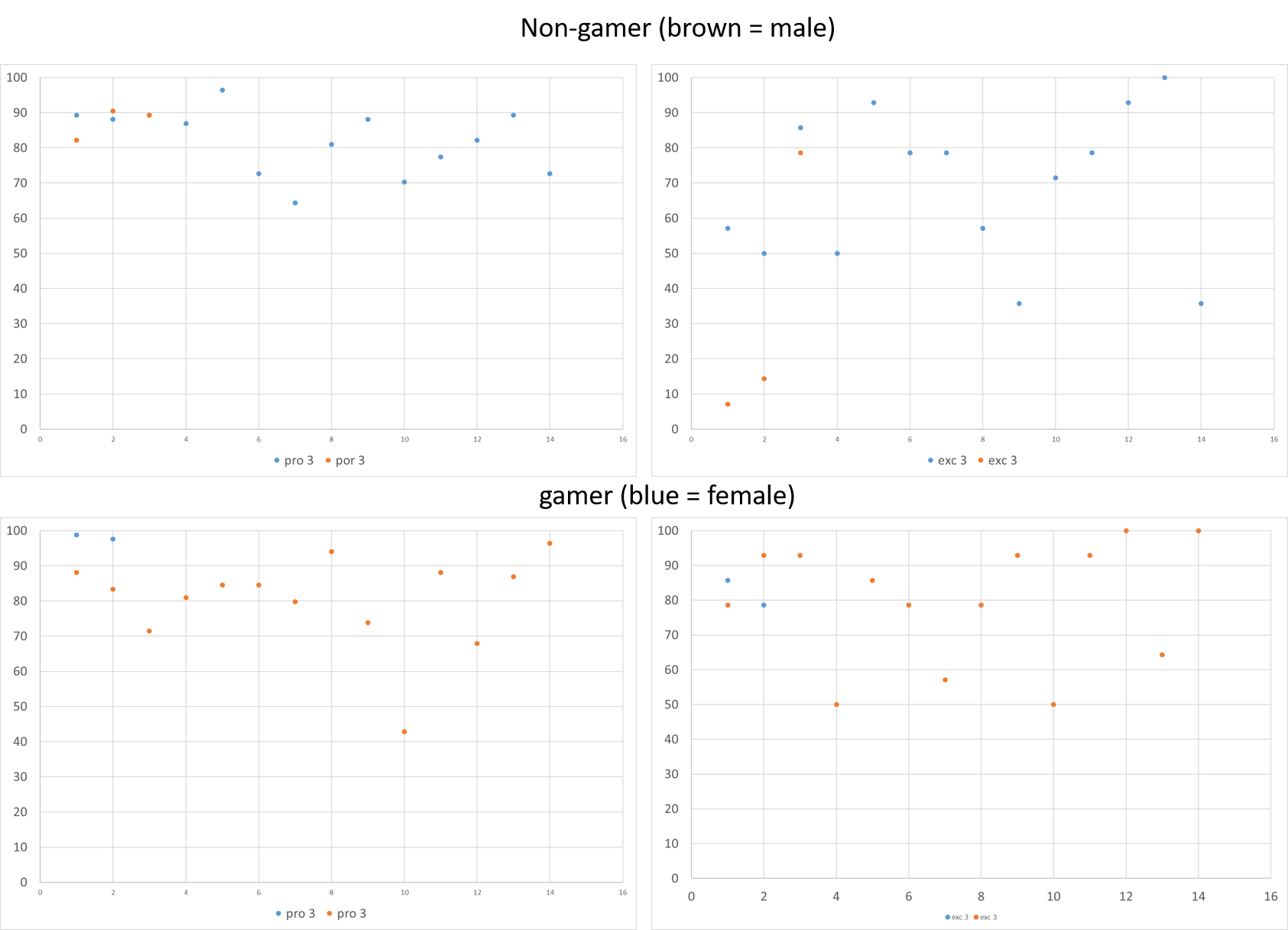

Supplement: Supplementary file 5 [file Image_3.TIF]

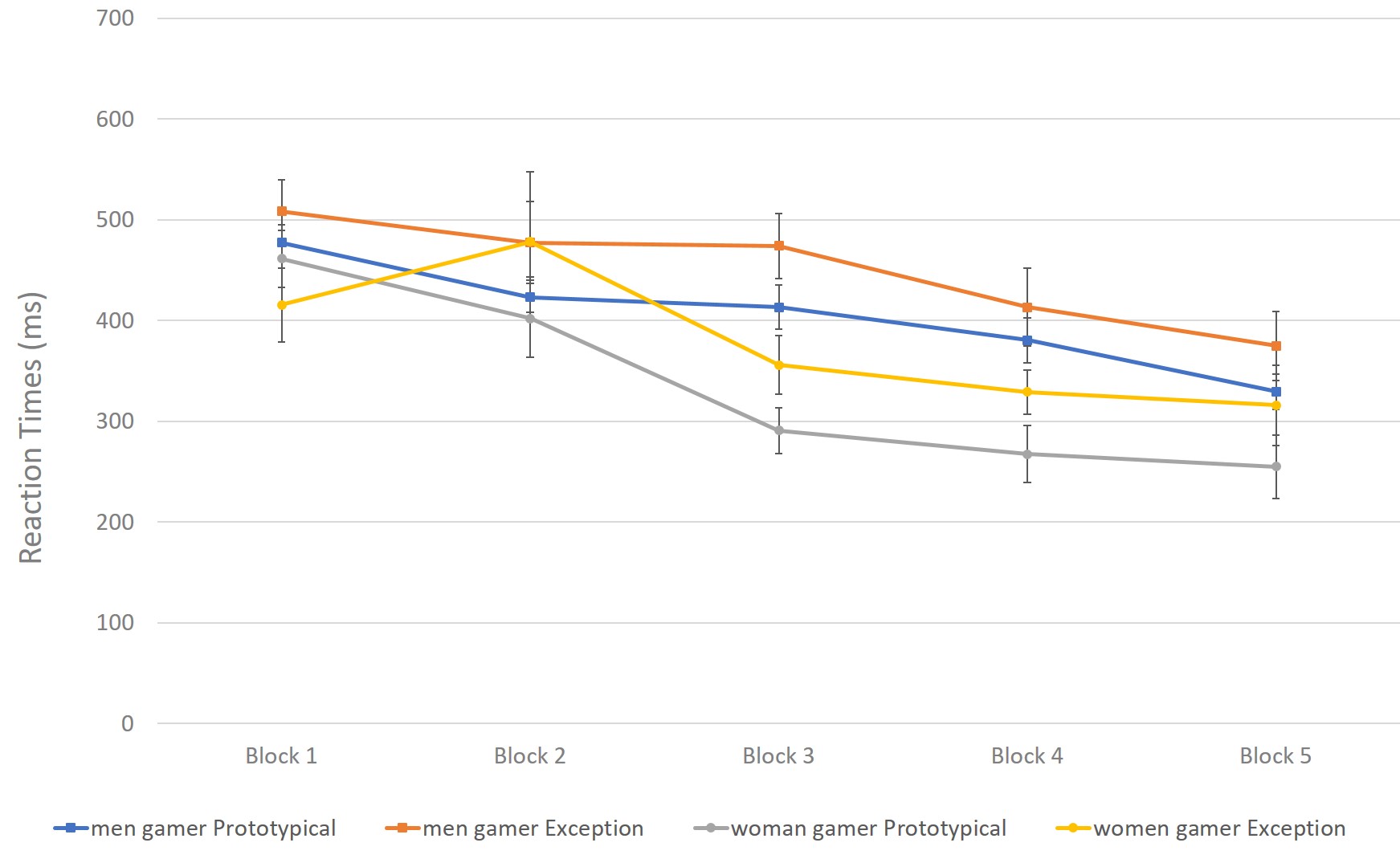

Supplement: Supplementary file 6 [file Image_4.JPEG]

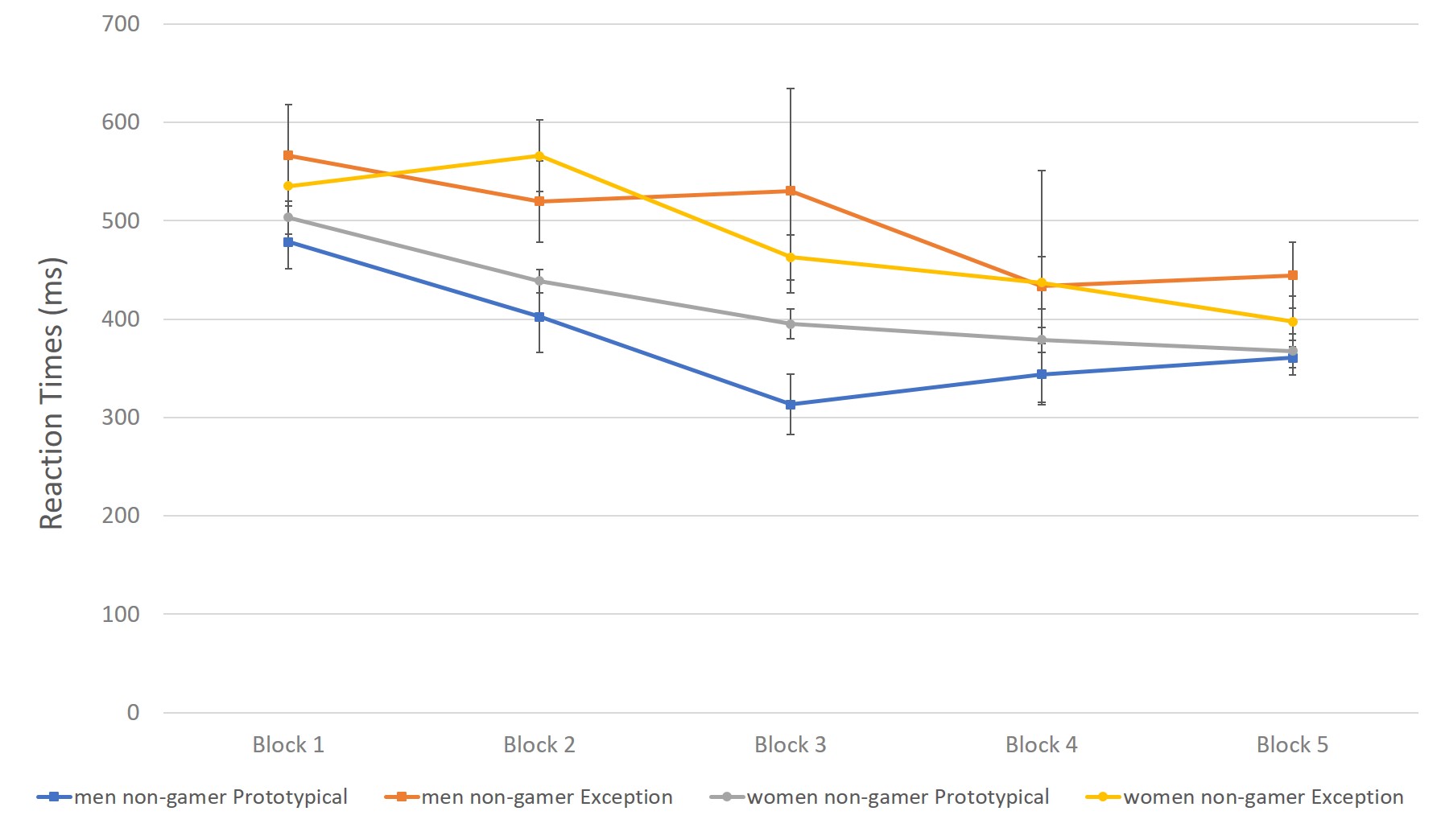

Supplement: Supplementary file 7 [file Image_5.JPEG]
